# Gender differences in health and health care utilisation in various ethnic groups in the Netherlands: a cross-sectional study

**DOI:** 10.1186/1471-2458-9-109

**Published:** 2009-04-20

**Authors:** Annette AM Gerritsen, Walter L Devillé

**Affiliations:** 1 Department of Public Health, University of Venda, Thohoyandou, South Africa and Netherlands Institute for Health Services Research (NIVEL), Utrecht, The Netherlands; 2 NIVEL, Utrecht, The Netherlands and University of Amsterdam, Medical Anthropology and Sociology Unit, Amsterdam, The Netherlands

## Abstract

**Background:**

To determine gender differences in health and health care utilisation within and between various ethnic groups in the Netherlands.

**Methods:**

Data from the second Dutch National Survey of General Practice (2000–2002) were used. A total of 7,789 persons from the indigenous population and 1,512 persons from the four largest migrant groups in the Netherlands – Morocco, Netherlands Antilles, Turkey and Surinam – aged 18 years and older were interviewed. Self-reported health outcomes studied were general health status and the presence of acute (past 14 days) and chronic conditions (past 12 months). And self-reported utilisation of the following health care services was analysed: having contacted a general practitioner (past 2 months), a medical specialist, physiotherapist or ambulatory mental health service (past 12 months), hospitalisation (past 12 months) and use of medication (past 14 days). Gender differences in these outcomes were examined within and between the ethnic groups, using logistic regression analyses.

**Results:**

In general, women showed poorer health than men; the largest differences were found for the Turkish respondents, followed by Moroccans, and Surinamese. Furthermore, women from Morocco and the Netherlands Antilles more often contacted a general practitioner than men from these countries. Women from Turkey were more hospitalised than Turkish men. Women from Morocco more often contacted ambulatory mental health care than men from this country, and women with an indigenous background more often used over the counter medication than men with an indigenous background.

**Conclusion:**

In general the self-reported health of women is worse compared to that of men, although the size of the gender differences may vary according to the particular health outcome and among the ethnic groups. This information might be helpful to develop policy to improve the health status of specific groups according to gender and ethnicity. In addition, in some ethnic groups, and for some types of health care services, the use by women is higher compared to that by men. More research is needed to explain these differences.

## Background

Gender differences in health and health care utilisation are well documented. Women generally experience poorer health than men, [1-5]although some studies have shown that the direction and magnitude of gender differences in health may vary according to the particular health outcome.[6,7] Determinants of gender differences in health include biological (e.g. genetic and hormonal factors), psychological (e.g. gender images and identities, chronic stressors), behavioural (smoking, drinking, eating, physical exercise) and social factors (e.g. social support, socio-economic status).[3,7,8] Research on patterns of health care utilisation suggests that, in general, women have higher utilisation rates of medical services than men, after controlling for health outcomes, [2,9] although differences might be small.[1] Various explanations for women's greater service use have been suggested: differences in social role, health knowledge, health status, sensitivity to symptoms, willingness to report health problems, acceptance of help seeking, compliance with treatment.[9]

Besides gender differences in health and health care utilisation, ethnic differences have also been the subject of research. In general, migrant groups experience a poorer health compared to that of the indigenous population. [10-15] This poorer health may result from circumstances including adverse socio-economic status, cultural factors (e.g. a different perception of health) or biological factors (e.g. genetic factors). Furthermore, differences in health care utilisation between migrant groups and the indigenous population have been reported, although these differences vary regarding the type of health care service (e.g. general practitioner, outpatient medical specialist, hospitalisation) and among migrant groups.[10,16-21] Differences can be partly explained by variation in age, gender, socio-economic position, health status and ethnicity. Factors which may restrict ethnic minority patients from using health services are complex and include such variables as health beliefs and attitudes, language, family and social support, physicians' skills and attitudes toward minority patients, and familiarity with the health care delivery system. [21,22]

Studies on ethnic differences in health and health care utilisation often adjust for gender and/or report results for women and men separately, without systematically reviewing gender differences in health and health care utilisation within and between ethnic groups.[10,11,13,15,18,19] However, some studies highlight the gender differences found, despite the fact that this was not the principal aim of the study.[12,14,17]

Only three studies were found to focus on gender differences in self-rated health,[23] functional limitations and life-threatening medical conditions[24], and depressive disorders [25] in various ethnic groups. And only one study examined gender differences in healthcare utilization across race/ethnicity. [26] Furthermore, no comparisons were made of gender differences between all ethnic groups studied, only comparisons of women and men within ethnic groups and/or comparisons with white men used as the reference category.

Little is known about whether gender disparities persist across different ethnic groups or whether they differ due to cultural differences, for example regarding the position of women in the society. Therefore the purpose of the current study is to determine gender differences in health and health care utilisation in various ethnic groups in the Netherlands. This information might be helpful to develop policy to focus on the health status and accessibility of the health care system of specific groups according to gender and ethnicity.

## Methods

### Study population

Data were derived from the second Dutch National Survey of General Practice, carried out between 2000 and 2002. A more extensive description of the methods of the survey, including the design, study population (response and representativeness) and data-collection can be found elsewhere.[27] The survey was carried out in a representative sample of 104 general practices in the Netherlands, constituting a cohort of 385,461 persons. All registered patients were requested to co-operate in a census to determine their socio-demographic characteristics (response 76.5%, 294,999 persons). Approximately 5% (19,685) of the Dutch-speaking registered persons, regardless of ethnic background, were randomly selected per practice and asked to participate in a face-to-face health interview (response 64.5%, 12,699 persons of 0–97 years old). Refusal was the most common reason for non-response (66.9%). In addition, interviews were held with individuals from the four largest migrant groups in the Netherlands: Morocco, the Netherlands Antilles, Turkey, and Surinam. If necessary, the interviews were held in the respondent's own language. The sample for this additional interview was drawn randomly from the census data that contains information on the ethnic background of people. Ethnic background was indicated by the country of birth of the person or their parents (i.e. when at least one parent was born abroad, the individual was recorded as having a foreign background).[28] 2,682 persons of 18 years and older were approached and 1,339 responded (49.9%). The most important reasons for non-response were difficulties in reaching the sampled persons (49.5%) and refusal (41.2%). However, in both health interview samples (Dutch-speaking persons and migrant groups) no differences in age, gender, socio-economic position and general health status (obtained from the census data) between respondents and non-respondents were found.

The final sample which is used in this article includes 9,301 persons of 18 years and older with the following ethnic background: 7,789 indigenous, 397 Moroccan, 284 Netherlands Antillean, 437 Turkish and 394 Surinamese.

### Outcome measures

Data on health and health care utilisation were obtained from the interviews. The current health status of the respondents was measured according to the general health question of the Short Form-36 (SF-36).[29] For the analyses the response options were dichotomised into good (excellent, very good, good) and (very) poor. Physical health was measured according to a list of 36 acute conditions (e.g. flu; ear ache; diarrhoea; headache; fatigue), and the respondents were asked to indicate whether or not they had had these illnesses in the previous 14 days or had suffered from an acute condition that was not included in the list (item 37).[30] A sum-score was calculated (0–37), and for the analyses the respondents were divided into two groups with '0 or 1' and 'more than 1' acute condition, respectively. Furthermore, a list of 19 chronic conditions (e.g. cardiovascular diseases; pulmonary diseases; diabetes) was included on which respondents had to indicate whether or not they had had this condition in the previous 12 months or had suffered from a condition that was not included in the list (item 20). A sum-score was calculated (range 0–20) and for the analyses dichotomised into '0' versus '1 or more' chronic condition(s).

To obtain an indication of the use of health care services, the following (self-reported) data were recorded:.[30] 1) contacts with a general practitioner in the previous 2 months; 2) contacts with an outpatient medical specialist in the previous 12 months; 3) hospital admissions (hospitalisation for one or more nights) in the previous 12 months; 4) contacts with a physiotherapist in the previous 12 months; 5) contacts with ambulatory mental health care (e.g. psychologist, psychiatrist) in the previous 12 months; 6) use of prescribed medication in the previous 14 days; and (7) use of over the counter medication in the previous 14 days. For the analyses, all data were dichotomised into 'no' versus 'any' use of health care services. This because comparisons of self-reported data with administrative data found that measures of any medical use were more accurate than measures of quantity, which were subject to underreporting. [31]

The following socio-demographic characteristics were obtained from the census data: ethnic background (indigenous, Morocco, Netherlands Antilles, Turkey or Surinam), gender, age and socio-economic position. The socio-economic position was indicated by the level of education (none/elementary, high school, college/university) and the type of insurance (public – which, in general, reflects a lower income – or private).

### Statistical analyses

First we examined differences in socio-demographic characteristics, health and health care utilisation between women and men for each ethnic group separately. For the socio-demographic characteristics this was conducted with two-tailed Pearson chi-square tests (education and insurance type) and Student's t-tests (age). For all health outcomes and utilisation of health care services univariate logistic regression models were used with gender as independent variable (men were used as the reference category).

Secondly, multivariate logistic regression was used to compare gender differences in health and health care utilisation within and between the various ethnic groups. For each outcome measure a model was fitted, containing gender and ethnic background and controlling for socio-demographic characteristics (age, education and insurance type). In the analyses concerning utilisation of health care services, the health outcomes (general health status, acute and chronic conditions) were added to the model to control for need. Per ethnic group gender differences in health and health care utilisation were expressed as (adjusted) odds ratios (ORs) with corresponding 95% confidence intervals (CIs). In addition, interaction terms for gender and ethnic background were included in all models, because the purpose of this explorative study was not only to examine discrepancies in gender differences between the migrant groups and the indigenous population, but also between the various migrant groups. A significant interaction (P < .05) indicates that in the two groups that are compared, the gender difference is not the same. Therefore, the multivariate logistic models were repeated, each time using a different ethnic group (including the indigenous population) as the reference category. All analyses were performed with SPSS (version 11).

## Results

Additional file 1 presents a table with the socio-demographic characteristics, reported health and health care utilisation of women and men per ethnic group and the gender differences within each ethnic group. Regarding the socio-demographic characteristics there were only few statistically significant differences (P < .05) between women and men. Among the respondents from Morocco the men were older than the women. In the Turkish group the women were less educated than the men. The difference in level of education was also statistically significant in the indigenous population. In the indigenous population the men were more often privately insured than the women.

To give an impression of the (unadjusted) gender differences in health and health care utilisation within ethnic groups the P-values are presented in the table of additional file 1. However, the significant differences between women and men are only discussed after reviewing Table 1 (adjusted analyses), to avoid unnecessary double information.

**Table 1:** Adjusted ORs (95% CIs) for gender differences in health and health care utilisation per ethnic group*

	**Indigenous** **(N = 7,789)**	**Morocco** **(N = 397)**	**Netherlands Antilles** **(N = 284)**	**Turkey** **(N = 437)**	**Surinam** **(N = 394)**
**Health**					

(Very) poor general health status	**1.14 [1.01–1.29]**	**1.95 [1.27–3.01]**	.92 [.53–1.59]	**2.72 [1.76–4.18]**	**2.02 [1.17–3.47]**

> 1 acute condition (14 days)	**1.89 [1.70–2.10]**	**2.87 [1.77–4.67]**	**2.01 [1.15–3.53]**	**4.48 [2.69–7.48]**	**1.97 [1.21–3.21]**

≥ 1 chronic conditions (12 months)	**1.53 [1.39–1.70]**	**2.33 [1.53–3.56]**	1.35 [.80–2.28]	**3.49 [2.30–5.30]**	**2.27 [1.40–3.68]**

**Health care utilisation**					

Contact general practitioner (2 months)	**1.29 [1.18–1.43]**	**1.73 [1.12–2.67]**	**1.99 [1.16–3.41]**	1.32 [.87–1.99]	.90 [.56–1.44]

Contact outpatient medical specialist (12 months)	1.07 [.97–1.19]	.97 [.61–1.53]	1.78 [.99–3.18]	1.15 [.73–1.83]	.73 [.45–1.20]

Hospitalisation (12 months)	1.00 [.83–1.19]	.98 [.44–2.21]	1.60 [.63–4.06]	**4.03 [1.50–10.80]**	1.28 [.46–3.55]

Contact physiotherapist (12 months)	**1.16 [1.01–1.32]**	.81 [.43–1.53]	1.31 [.58–2.97]	1.14 [.63–2.07]	.57 [.31–1.03]

Contact ambulatory mental health care (12 months)	**1.43 [1.17–1.76]**	**2.92 [1.16–7.36]**	1.03 [.47–2.22]	.81 [.43–1.55]	2.36 [.79–7.06]

Use of prescribed medication (14 days)	1.10 [.98–1.22]	1.29 [.78–2.15]	1.06 [.57–1.95]	.98 [.61–1.58]	.89 [.53–1.52]

Use of over the counter medication (14 days)	**1.68 [1.52–1.85]**	1.43 [.91–2.25]	1.23 [.72–2.09]	.96 [.62–1.47]	1.26 [.77–2.07]

* Men are the reference category.

Adjusted for age, education and insurance type. Utilisation of health care services also

adjusted for general health status, acute and chronic conditions.

Statistically significant gender differences within ethnic groups (P < .05) are printed in bold.

In Table 1 the ORs for gender differences in health and health care utilisation are presented for all groups, adjusted for age, education and insurance type, and also for the health outcomes (general health status, acute and chronic conditions) in case of utilisation of health care services.

When reviewing the gender differences within ethnic groups, the health of women was worse compared to that of men, except for respondents from the Netherlands Antilles. For Antilleans this was only true for acute conditions. For general health status and chronic conditions no statistically significant differences (P < .05) between women and men from the Antilles could be found.

For use of health care the results were less consistent and, in general, for most services no (significant) gender differences were observed. Women from Morocco and the Netherlands Antilles more often contacted a general practitioner than men from these countries. Turkish women were more often hospitalised than Turkish men. Women with a Moroccan background more often contacted ambulatory mental health care. Although this gender difference is also large for the Surinamese group, it was not statistically significant. Finally, in the indigenous group women more often used over the counter medication than men. For the indigenous population the gender difference for contact with a general practitioner, physiotherapist and ambulatory mental health care were also statistically significant.

In the multivariate logistic regression models interaction terms for gender and ethnic background were included to examine differences between the ethnic groups. Only the ethnic groups for which there was a gender difference within the group and for which the OR for this gender difference was not the same compared to the OR of another group, according to significant interaction terms (P < .05), are mentioned.

Regarding general health status the ORs of the Turks (2.72), Surinamese (2.02) and Moroccans (1.95) are significantly higher than those of the indigenous population (1.14) and Antilleans (0.92). For acute and chronic conditions the OR of the Turkish group (4.48 and 3.49 respectively) is higher than those of the indigenous population (1.89 and 1.53) and Antilleans (2.01 and 1.35), and for acute conditions also higher than the OR of the Surinamese group (1.97). The ORs of the Moroccans (1.73) and Antilleans (1.99) are significantly higher than that of the Surinamese (.90) with respect to contact with a general practitioner. For hospitalisation the OR of the Turks (4.03) is significantly higher than those of the indigenous population (1.00) and Moroccans (.98). The OR of the Moroccan group (2.92) is significantly higher than that of the Turkish group (.81) for contact with ambulatory mental health care. And the OR of the indigenous population (1.68) is significantly higher than that of the Turkish population (.96) regarding use of over the counter medication.

## Discussion

The purpose of the analyses presented in this paper was to determine gender differences in health and health care utilisation within and between various ethnic groups in the Netherlands. This information might be helpful to develop policy to focus on the health status and accessibility of the health care system of specific groups according to gender and ethnicity.

In general women showed poorer health than men; the largest differences were found for the Turkish respondents, followed by Moroccans, and Surinamese. Furthermore, women from Morocco and the Netherlands Antilles more often contacted a general practitioner than men with this background. Women from Turkey were more hospitalised than Turkish men. Women from Morocco more often contacted ambulatory mental health care than men with this background, and women with an indigenous background more often used over the counter medication than men with an indigenous background.

It turned out to be difficult to obtain estimates of gender differences for the different ethnic groups in their countries of origin. A study of service utilization in Curaçao, Netherlands Antilles, showed that women were more likely to consult a general practitioner or specialist than men, which is in agreement with our findings (although the specialist consultation was borderline significant). [32] Furthermore, nationwide surveys of the Ministry of Health of Turkey indicate that the health status of women is poorer (self perceived health status and chronic diseases) than that of men and that there is a need to study the underlying psycho-social causes of this situation.[33] They furthermore show that women are hospitalized more than men. Both findings are in agreement with the findings in the current study.

In the literature only four studies were found which also focused on gender differences in health in various ethnic groups. One study examined inequalities in the self-reported health of men and women from white and minority ethnic groups in the UK.[23] The results showed higher morbidity (i.e. worse general health status) for women from the Black Caribbean and Indian populations, but not for women from the white (indigenous), Pakistani and Bangladeshi populations. Furthermore, the analyses showed substantially poorer health among all minority ethnic groups (men and women) compared to white men. Another study assessed the association between gender, race/ethnicity (white, moreno, mulatto, black), and social class and prevalence of depressive disorders in an urban sample in Brazil.[25] The study showed that there was no female:male difference in depression among Whites, and that the highest ORs for gender difference were found in the moreno and black ethnic group (adjusted for i.e. social class). A recently conducted study assessed differences in men's and women's self-rated health, functional limitations and life-threatening medical conditions across five major US racial/ethnic populations.[24] The results showed that the magnitude of gender differences varies considerably by racial/ethnic group, health outcome, and comparison category. When compared to white men, Non-Hispanic blacks (men and women), Mexican women, Puerto Ricans (men and women) and Cuban women are more likely than white men to report fair/poor general health status when adjusted for demographic and socio-economic factors. Finally, a study examining gender differences in health care utilization among older Americans found that gender differences in medical use vary according to the type of services used: women are less likely to use hospitalization and outpatient surgery but are more likely to use physician and home health services than men. [26] These differences are largely consistent in direction and magnitude across racial/ethnic groups (white, African American, Hispanic).

The results of the current study regarding gender differences within ethnic groups are in agreement with the study conducted in the UK, showing that the general health status of women is worse compared to that of men in some groups (e.g. the Moroccan, Turkish and Surinamese groups) and that the gender differences are small or absent in other groups (e.g. the indigenous group and the Antillean group, respectively). Regarding health care utilization, the results differ somewhat from the study conducted among older Americans, as gender differences in health care use vary among the different ethnic groups, although differences are in general small and there is no clear trend. Based on the results of the current study we can not conclude that gender differences in health and health care utilization among the ethnic groups clearly vary due to cultural differences, for example regarding the position of women in the society. Other studies should be conducted to explain the gender differences between the ethnic groups, by focussing on cultural, but also on e.g. biological and socio-economic factors.

### Strengths and limitations

The non-response rate in the migrant groups was higher than in the indigenous population. This was mainly due to difficulties in reaching the sampled persons, a well-known problem in population-based studies among migrants.[10,18] It is not clear whether the results of the study were consequently biased. However, no differences in age, gender, socio-economic position and general health status (obtained from the census data) between respondents and non-respondents were found.

There is the possibility of bias from using self-reported data. However, general health status has been extensively used in surveys all over the world and the outcome measures on acute and chronic conditions are included in the surveys of Statistics Netherlands,.[30] although evidence on their cross-cultural validity is limited. Reporting of health and use of health care might differ between women and men, although women are not per se more willing to report symptoms than men.[6] Self-reported data turned out to be a useful method for providing a valid estimation of ethnic differences in health care utilisation.[14,34] In order to restrict bias as much as possible the comprehensibility and acceptability of the questionnaire was tested in a pilot. Furthermore, interviews were held in the respondent's own language if necessary.

Given the large sample size of the indigenous population, some of the gender differences in health and health care utilisation are statistically significant (e.g. level of education, general health status, contact with a general practitioner, physiotherapist and ambulatory mental health care), although some were small and not socially or biologically relevant. On the other hand, the numbers of respondents in the migrant groups are relatively small, yielding broad confidence intervals for the ORs (e.g. for general health status and chronic conditions in Antilleans, ambulatory mental health care in Surinamese). Therefore, P < .05 was used for the multivariate analyses, despite multiple testing. This was also justified because of the explorative nature of the study.

Gender and ethnic differences in health and health care have shown to be related to age and socio-economic position.[23,24] Consequently, the multivariate models also contained these variables. However, one could argue whether controlling for education and insurance type is sufficient to cover socio-economic position. Especially for the few more educated migrants, level of education might not be a good indicator, as issues such as being Muslim might make them less employable and therefore poorer compared to their indigenous equivalents, hence leading to worse health outcomes.

The strengths of the current study include the relatively large sample sizes of the migrant groups compared to other studies on health and health care utilisation among these groups conducted in the Netherlands,[10,19] and the use of several health outcomes and particularly the use health care utilisation rates compared to other studies on gender and ethnicity. [23,24]

Furthermore, a recent study that addresses the importance of the need to integrate a gender perspective into epidemiological studies on migration and health states that information on both ethnic background and sex, together with socio-economic status is not usually available in most health information systems. [35] All these variables are included in the survey data on which the current study is based.

## Conclusion

The current study allows the exploration of how health and health care utilisation differs between women and men in various ethnic groups in the Netherlands.

In general the self-reported health of women is worse compared to that of men, although the size of the gender differences may vary according to the particular health outcome and among the ethnic groups. This information might be helpful to develop policy to improve the health status of specific groups according to gender and ethnicity. In addition, in some ethnic groups, and for some types of health care services, controlled for health status the use by women is still higher compared to that by men. It could be over-utilisation of health care by women or under-utilisation by men. However, general-purpose surveys, such as the second Dutch National Survey of General Practice from which the data were derived, have limited utility in assessing explanations for the differences found. Future research should therefore have to focus more in depth on gaining insight into determinants of gender difference in health and health care utilisation between various ethnic groups.

## Competing interests

The authors declare that they have no competing interests.

## Authors' contributions

AAMG analysed and interpreted the data, drafted the manuscript and has given final approval of the version to be published. WLD was involved in the collection of the data, interpreted the data, critically revised the draft of the manuscript and has given final approval of the version to be published.

## Pre-publication history

The pre-publication history for this paper can be accessed here:



## Supplementary Material

Additional file 1 **Supplementary table**. Socio-demographic characteristics, health and health care utilisation of women and men per ethnic group. Click here for file
