# Supplementary material for: Gender differences in health and health care utilisation in various ethnic groups in the Netherlands: a cross-sectional study
Source: BMC Public Health. 2009 Apr 20;9:109. doi: 10.1186/1471-2458-9-109 (PMC2678118; doi:10.1186/1471-2458-9-109)
Supplement: Additional file 1 — Supplementary table. Socio-demographic characteristics, health and health care utilisation of women and men per ethnic group. [file 1471-2458-9-109-S1.doc]

Additional file 1: Socio-demographic characteristics, health and health care utilisation of women and men per ethnic group*

|  | Indigenous **(N=7,789)** | | | **Morocco**  **(N=397)** | | | **Netherlands Antilles**  **(N=284)** | | | **Turkey**  **(N=437)** | | | **Surinam**  **(N=394)** | | |
| --- | --- | --- | --- | --- | --- | --- | --- | --- | --- | --- | --- | --- | --- | --- | --- |
|  | Women  (n=4,291) | Men  (n=3,498) | P | Women  (n=227) | Men  (n=210) | P | Women  (n=282) | Men  (n=112) | P | Women  (n=208) | Men  (n=189) | P | Women  (n=178) | Men  (n=106) | P |
| **Socio-demographic characteristics** |  |  |  |  |  |  |  |  |  |  |  |  |  |  |  |
| Age, mean (SD), years | 50.0  (17.2) | 49.6  (16.8) | .255 | 36.0 (13.0) | 37.0 (11.9) | .384 | 44.7 (14.8) | 44.8 (14.5) | .925 | 32.8 (11.6) | 39.7 (13.1) | .000 | 38.7  (13.7) | 40.4  (14.1) | .329 |
| Education  - None/elementary  - High school  - College/  university | 20.2  60.8  19.1 | 15.0  59.9  25.1 | .000 | 53.7  39.0  7.3 | 37.1  53.7  9.3 | .003 | 25.8  58.7  15.5 | 16.4  62.7  20.9 | .101 | 47.2  44.7  8.0 | 48.6  39.3  12.0 | .331 | 17.2  64.5  18.3 | 16.8  73.3  9.9 | .158 |
| Insurance type  - Public | 72.6 | 60.7 | .000 | 95.2 | 92.8 | .304 | 84.9 | 78.4 | .119 | 95.7 | 93.0 | .261 | 85.3 | 76.7 | .070 |
| **Health** |  |  |  |  |  |  |  |  |  |  |  |  |  |  |  |
| (Very) poor general health status | 19.1 | 15.8 | .000 | 44.9 | 23.8 | .000 | 35.5 | 21.6 | .008 | 43.0 | 32.8 | .037 | 30.9 | 34.0 | .593 |
| > 1 acute condition (14 days) | 80.6 | 67.9 | .000 | 89.0 | 64.8 | .000 | 76.5 | 64.3 | .014 | 83.2 | 64.0 | .000 | 78.7 | 63.2 | .005 |
| ≥ 1 chronic conditions (12 months) | 70.0 | 60.3 | .000 | 73.1 | 45.2 | .000 | 72.7 | 57.1 | .003 | 64.4 | 49.7 | .003 | 60.7 | 55.7 | .406 |
| Health care utilisation |  |  |  |  |  |  |  |  |  |  |  |  |  |  |  |
| Contact general practitioner (2 months) | 46.1 | 36.6 | .000 | 60.6 | 43.3 | .000 | 54.6 | 50.5 | .457 | 55.1 | 37.6 | .000 | 55.1 | 37.7 | .005 |
| Contact outpatient medical specialist (12 months) | 43.1 | 38.8 | .000 | 63.0 | 51.5 | .028 | 54.4 | 53.8 | .927 | 47.6 | 42.1 | .294 | 63.3 | 50.5 | .048 |
| Hospitalisation (12 months) | 7.6 | 6.8 | .171 | 12.4 | 2.4 | .000 | 7.1 | 4.5 | .350 | 6.8 | 6.3 | .868 | 10.7 | 6.7 | .254 |
| Contact physiotherapist (12 months) | 16.8 | 13.0 | .000 | 17.2 | 10.0 | .029 | 18.1 | 19.6 | .720 | 13.5 | 11.6 | .585 | 12.4 | 9.4 | .451 |
| Contact ambulatory mental health service (12 months) | 7.4 | 4.7 | .000 | 12.3 | 9.0 | .268 | 11.0 | 4.5 | .042 | 13.9 | 3.2 | .000 | 14.0 | 12.3 | .670 |
| Use of prescribed medication (14 days) | 50.1 | 43.6 | .000 | 44.1 | 31.9 | .009 | 54.0 | 47.3 | .235 | 39.1 | 33.0 | .204 | 41.0 | 40.6 | .941 |
| Use of over the counter medication (14 days) | 44.3 | 29.9 | .000 | 33.0 | 29.0 | .368 | 39.7 | 30.4 | .084 | 38.2 | 24.1 | .003 | 42.7 | 33.0 | .106 |

* Percentage of respondents is presented, unless indicated otherwise.
